# Inactivation of PI3-K/Akt and reduction of SP1 and p65 expression increase the effect of solamargine on suppressing EP4 expression in human lung cancer cells

**DOI:** 10.1186/s13046-015-0272-0

**Published:** 2015-12-21

**Authors:** YuQing Chen, Qing Tang, JingJing Wu, Fang Zheng, LiJun Yang, Swei Sunny Hann

**Affiliations:** Laboratory of Tumor Biology, Department of Medical Oncology, Guangdong Provincial Hospital of Chinese Medicine, The Second Clinical Medical Collage, University of Guangzhou Traditional Chinese Medicine, Guangzhou, Guangdong Province 510120 China; Higher Education Mega Center, No. 55, Neihuan West Road, Panyu District, Guangzhou, Guangdong Province 510006 PR China

**Keywords:** Human lung cancer cells, Solamargine, PI3-K/Akt, SP1, EP4, p65

## Abstract

**Background:**

Lung cancer is the most common cause of cancer-related deaths worldwide. Natural phytochemicals from traditional medicinal plants such as solamargine have been shown to have anticancer properties. The prostaglandin E2 receptor EP4 is highly expressed in human cancer, however, the functional role of EP4 in the occurrence and progression of non small cell lung cancer (NSCLC) remained to be elucidated.

**Methods:**

Cell viability was measured by MTT assays. Western blot was performed to measure the phosphorylation and protein expression of PI3-K downstream effector Akt, transcription factors SP1, p65, and EP4. Quantitative real-time PCR (qRT-PCR) was used to examine the mRNA levels of EP4 gene. Exogenous expression of SP1, p65, and EP4 genes was carried out by transient transfection assays. EP4 promoter activity was measured by Dual Luciferase Reporter Kit.

**Results:**

We showed that solamargine inhibited the growth of lung cancer cells. Mechanistically, we found that solamargine decreased the phosphorylation of Akt, the protein, mRNA expression, and promoter activity of EP4. Moreover, solamargine inhibited protein expression of SP1 and NF-κB subunit p65, all of which were abrogated in cells transfected with exogenous expressed Akt. Intriguingly, exogenous expressed SP1 overcame the effect of solamargine on inhibition of p65 protein expression, and EP4 protein expression and promoter activity. Finally, exogenous expressed EP4 feedback reversed the effect of solamargine on phosphorylation of Akt and cell growth inhibition.

**Conclusion:**

Our results show that solamargine inhibits the growth of human lung cancer cells through inactivation of Akt signaling, followed by reduction of SP1 and p65 protein expression. This results in the inhibition of EP4 gene expression. The cross-talk between SP1 and p65, and the positive feedback regulatory loop of PI3-K/Akt signaling by EP4 contribute to the overall responses of solamargine in this process. This study unveils a novel mechanism by which solamargine inhibits growth of human lung cancer cells.

## Background

Lung cancer is the most common cancer and the leading cause of cancer mortality worldwide for both men and women [1]. Most patients present with incurable advanced or metastatic disease with poor 5-year survival rate. Among them, more than 80 % of lung cancers are non-small cell lung carcinoma (NSCLC) with adenocarcinoma as the most prevalent subtype [1]. The choice of treatment for patients with advanced disease remains dilemma and challenge, which dependent on the histological types, tumor characteristics, stages, co-morbidities and prior therapies history. Inspire of the advance in understanding the molecular mechanism and treatment options, the poor patient survival still remain no changes and the caused debilitating symptoms seriously affect the quality of life of patients. Therefore, searching for more effective alternative treatment strategies in order to strengthen the therapeutic efficacy with negligible side effects is urgently needed.

Natural compounds, known as phytochemicals, obtained from traditional medicinal plants have gained more attention in the prevention and intervention of human illness including cancers [2]. solamargine (SM), the typical metabolites of solanum lycocarpum fruit glycoalkaloid extract from traditional herbal medicine, demonstrated not only anti-viral, anti-inflammatory but also antiproliferative activity against the several types of human cancers including lung [3–7]. Report showed that SM induced apoptosis and inhibited growth of hepatoma SMMC-7721 cells through activation and induction expression of caspase-3 [8]. Early study found that combination of low concentrations of SM with low-toxic topoisomerase II inhibitor epirubicin synergistically accelerated apoptotic cell death through up-regulation of Fas expression and down-regulated the expression of human epidermalgrowth factor receptor-2 (HER2) and topoisomerase II alpha (TOP2A) in NSCLC A549 and H441 cells [9] However, the detailed mechanisms and potential therapeutic benefices by which this agent in controlling the growth of human lung cancer cells have not been well determined.

As a central to multiple signaling pathways, the phosphatidylinositol-3-kinase (PI3-K)/protein kinase B (Akt) has been shown to play an important role in a wide variety of fundamental cellular processes, such as proliferation, migration, apoptosis, metabolic homeostasis, hypoxia, and recently immune-regulation. Aberrant regulation of the PI3-K/Akt signaling axis is considered as a primary causative node in major diseases including cancer. It is therefore not surprising that this signaling pathway is frequently dysregulated in cancers, making the Akt important therapeutic target [10–13]. Activation of this pathway can be the result of mutations or amplification of PI3-K/Akt itself, which is frequently observed in multiple cancer types, such as NSCLC [14]. On the contrary, blockade of PI3K/Akt and other signaling pathways have been involved in the anticancer activities in several cancer types including lung [13, 15–17]. One report showed that the combination of BGT226, a novel PI3K/ mammalian target of rapamycin (mTOR) dual inhibitor, and gefitinib, one of epidermal growth factor receptor (EGF-R) tyrosine kinase inhibitors (TKIs), had synergistically inhibitory effects in growth of NSCLC cells through inhibition of PI3K/Akt/mTOR signaling pathways [18].

The E-prostanoid receptor 4 (EP4) is one of four receptor subtypes for prostaglandin E_2_ (PGE_2_), the key lipid mediators produced from arachidonic acid. It belongs to the family of G protein-coupled receptors and plays an important role in various cellular processes, such as inflammatory, angiogenesis, cancer growth, and progression [19]. A possible explanation for the diverse biological functions of EP4 might be the multiple signaling pathways switched on upon activation and increased expression of EP4 gene. A large body of evidence demonstrated that activation and/or high expression of EP4 have been shown in several malignancies and associated with growth and progression [19–23]. However, the functional significance of EP4 expression in lung cancer occurrence and progression remains to be elucidated.

In this study, we explored the potential mechanism by which solamargine inhibits growth of human lung cancer cells.

## Methods

### Cell culture and chemicals

The human cancer cancer lines A549 and H1299 were obtained from the (Sun Yat-sen Memorial Hospital, Sun Yat-sen University) and the Chinese Academy of Sciences Cell Bank of Type Culture Collection (Shanghai, China). All cell lines have been tested and authenticated for absence of Mycoplasma, genotypes, drug response, and morphology in the Laboratory since 2012. Cells were grown in RPMI 1640 medium (obtained from GIBCO, Life Technologies, Grand Island, NY, USA) with supplemented 10 % fetal bovine serum. Lipofectamine 3000 reagent was purchased from Invitrogen (Shanghai, China). The polyclonal antibody against EP4 was obtained from Abcam (Cambridge, MA, USA). The antibodies against SP1, total Akt and the phosphor-form (Ser473) were purchased from Cell Signaling Technology Inc (Beverly, MA, USA). Other chemicals unless indicated were obtained from Sigma-Aldrich (St. Louis, MO, USA).

### Cell viability assay

Cell viability was measured using the 3-(4, 5-dimethylthiazol-2-yl)-2, 5-diphenyltetrazolium bromide (MTT) assay [24]. In brief, NSCLC cells were harvested, counted, and seeded in a 96-well microtitre plate. The cells were treated with increasing concentrations of solamargine for up to 72 h. After incubation, 20 μL MTT solution (5 g/L) was added and incubated at 37 °C for an additional 4 h. afterwards, the supernatant was removed, followed by adding solvent dimethyl sulfoxide (200 μL) to each well and oscillated for 8 min. Finally, the absorbance of optical density at 490 nm was determined by ELISA reader (Perkin Elmer, Victor X5, USA). Cell viability (%) was calculated as (absorbance of test sample/absorbance of control) × 100 %.

### Cell cycle analysis

This procedure was reported previously [24]. In brief, NSCLC cells were cultured in 6-well plates at 1 × 10^5^ cells/well and treated with increased doses of solamargine for 24 h. Afterwards, the cells were harvested and resuspended in 500 μL of cold PBS and cold ethanol (1.5 mL) for 2 h at 4 °C, followed by incubating with 0.1 % sodium citrate containing propidium iodide (PI) 0.05 mg and 50 μg RNase for 30 min. Finally, the cell cycle analysis was detected by flow cytometry (FC500, Beckman Coulter, FL, USA), and the proportion of cells within the G0/G1, S, and G2/M phases of the cell cycle were analyzed using the MultiCycle AV DNA Analysis software (Phoenix Flow Systems).

### Transient transfection assay

The control, Akt, SP1, and EP4 overexpression vectors (pCMV6-AC-Akt, pCMV6-AC-SP1, pCMV6-AC-EP4) were obtained from OriGene Technologies, Inc. (Rockville, MD, USA). The p65 overexpression construct (pCMV4-p65) was obtained from the Addgene (Plasmid #21966) [25]. Briefly, cells were seeded in 6-well dishes and grown to 50–60 % confluence. For each well, 2 μg of control, SP1, p65 and EP4 plasmid DNA constructs were transfected into the cells using Lipofectamine 3000 reagent (Invitrogen, Shanghai, China) for 30 h, followed by treating with solamargine for an additional 24 or 48 h. In the separated experiments, control and wild type EP4 promoter constructs, a gift from Dr. Thomas Eling (NIEHS, USA), which was reported previously [26], with or without 0.25 μg of the internal control pRL-CMV Renilla luciferase reporter DNA (0.02 μg/well) were co-transfected into the cells with Lipofectamine 3000 Transfection Reagent (Invitrogen, Shanghai, China). The preparation of cell extracts and measurement of luciferase activities were determined using the Dual-Luciferase Reporter kit (Promega, Beijing, China). Firefly luciferase activity was normalized with Renilla luciferase activity within each sample.

### Western blot analysis

The detailed procedure was reported previously [2, 24]. Briefly, equal amounts of protein from whole cell lysates were solubilized in 5 × SDS-sample buffers and separated on 10-12 % SDS polyacrylamide gels. Membranes were incubated with antibodies against EP4, p65, SP1, the phosphor and total Akt. The membranes were washed and incubated with a secondary antibody raised against rabbit IgG conjugated to horseradish peroxidase (Cell Signaling Technology, Inc., Beverly, MA, USA). The membranes were washed again and transferred to freshly made ECL solution (Immobilon Western; Millpore, Billerica, MA, USA), followed by observing the signals under the Molecular Imager ChemiDoc XRS Gel Imagine System (Bio-Rad, Hercules, CA, USA) and documenting the results.

### Quantitative real-time-PCR

A quantitative real-time-PCR (qRT-PCR) assay was performed for the detection of EP4 transcripts. The primers used in this study were designed as follows: EP4 forward 5’- TCGCGCAAGGAGCAGAAGGACAC -3’; reverse 5’- GACGGTGGCGAGAATGAGGAAGGA -3’ [26]; GAPDH forward 5’- AAGCCTGCCGGTGACTAAC -3’; reverse 5’- GCGCCCAATACGACCAAATC -3’, which was used as an internal control. First-strand cDNA was synthesized from total RNA (2 μg) by reverse transcription using oligo-dT primers and Superscript II reverse transcriptase (Invitrogen, Grand Island, NY, USA) according to the manufacturer’s instructions. qRT-PCR was performed in a 20 μL mixture containing 2 μL of the cDNA preparation, 10 μL 2X SYBR Green Premix ExTaq (Takara), and 10 μM primer on an ABI 7500 Real-Time PCR System (Applied Biosystems, Grand Island, NY, USA). The PCR conditions were as follows: 10 min at 95 °C, followed by 40 cycles of 15 s at 95 °C, and 1 min at 60 °C. Each sample was tested in triplicate. Threshold values were determined for each sample/primer pair, the average and standard errors were calculated.

### Statistical analysis

All experiments were repeated a minimum of three times. All data were expressed as means ± SD. Differences between groups were assessed by one-way ANOVA and significance of difference between particular treatment groups was analyzed using Dunnett’s multiple comparison tests (GraphPadPrism5.0 software, LaJolla, CA). Asterisks shown in the figures indicate significant differences of experimental groups in comparison with the corresponding control condition (*P* < 0.05, see figure legends).

## Results

### The effect of solamargine on growth of lung cancer cells

**Fig. 1:**
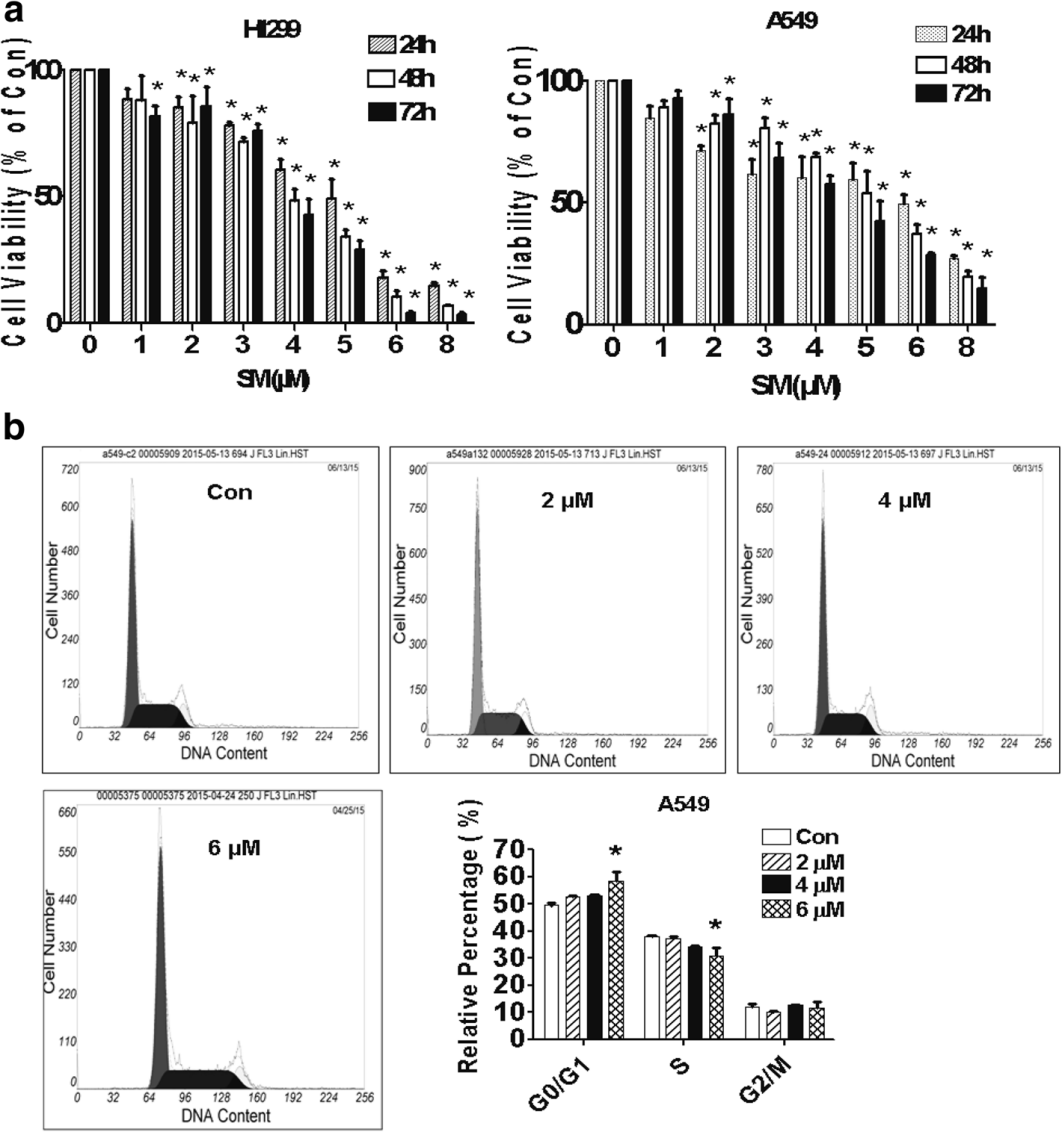
The effect of solamargine on growth of lung cancer cells. **a** Lung cancer cell lines (H1299 and A549) were treated with increased concentrations of solamargine for up to 72 h. Afterwards, the cell viability was determined using the MTT assay as described in the Materials and Methods section. **b** A549 cells were treated with increased concentrations of solamargine for up to 48 h. Afterwards, the cells were collected and processed for analysis of cell cycle distribution by flow cytometry after propidium iodide (PI) staining. The percentages of the cell population in each phase (G0/G1, S and G2/M) were assessed by Multicycle AV DNA Analysis Software. Values are given as the mean ± SD from 3 independent experiments performed in triplicate and expressed as a percentage of total cells. *indicates significant difference as compared to the untreated group (*P* < 0.05). **Indicates significant difference from solamargine treated alone (*P* < 0.05)

We first examined the effect of solamargine on lung cancer cell growth. We showed that solamargine inhibited the growth of H1299 and A549 lung cancer cells in the dose-dependent manner with the most significant effect observed at 6 μM for up to 72 h (Fig. 1a). Note that low doses also showed significant response in H1299 and A549 lung cancer cells. The IC50 were 4.09 and 3.40 μM in H1299 and A549 cells, respectively. We also performed the cell cycle experiment. Our results found that, compared with the untreated control cells, solamargine significantly increased the proportion of cells at G0/G1 phase, while the proportion of cells at S phase were reduced at the 6 μM solamargine (Fig. 1b) suggesting that solamargine induced cell cycle arrest in G0/G1 phase in lung cancer cells.

### Solamargine decreased phosphorylation of Akt

**Fig. 2:**
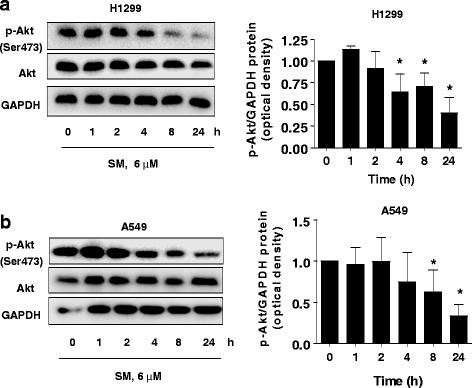
Solamargine decreased phosphorylation of Akt. **a**-**b** H1299 and A549 cells were treated with solamargine (6 μM) in the indicated times, and cell lysate was harvested and the expression of the phosphorylated and total protein of Akt was measured by Western blot. GAPDH was used as loading control. Values in bar graphs were given as the mean ± SD from three independent experiments. *indicates significant difference as compared to the untreated control group (*P* < 0.05)

We then explored the signaling pathways that were involved in the inhibitory response by solamargine in lung cancer cells. We showed that solamargine decreased the phosphorylation of Akt (ser473), a downstream effector of PI3-K, in a time-dependent fashion with significant reduction observed at 4-24 h in H1299 and A549 cells (Fig. 2a-b).

### Solamargine inhibited EP4 mRNA, protein and promoter activity

**Fig. 3:**
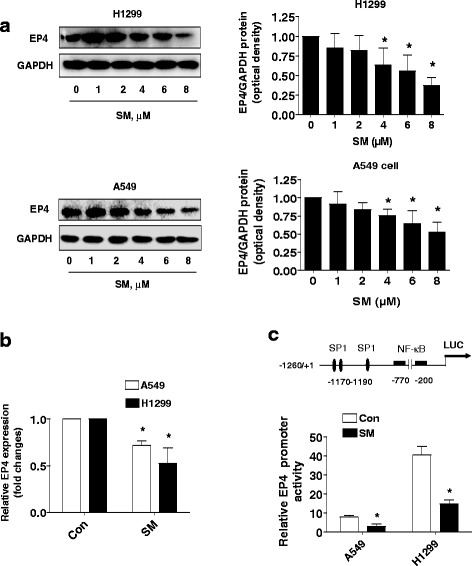
Solamargine inhibited EP4 mRNA, protein and promoter activity. **a** H1299 and A549 cells were exposed to increased concentration of solamargine for 24 h. Afterwards, the expression of EP4 proteins was detected by Western blot. **b** H1299 and A549 cells were treated with A549 and H1975 cells were exposed to solamargine (6 μM) for 24 h, followed by measuring the mRNA levels by qRT-PCR. **c** A549 and H1975 cells were transfected with a wild type human EP4 promoter reporter construct ligated to luciferase reporter gene and internal control for 24 h, followed by exposing to solamargine for an additional 24 h. Afterwards, the promoter activities of EP4 were determined using the Dual-Luciferase Reporter kit (Promega), as described in the Materials and Methods section. Values in bar graphs were given as the mean ± SD from three independent experiments performed in triplicate. *indicates significant difference as compared to the untreated group (*P* < 0.05)

Next, we examined the potential molecular targets by solamargine. PGE_2_ receptor EP4 has been shown to be associated with tumor growth and progression [19–22]. We showed that solamargine reduced the protein, mRNA expression, and promoter activity of EP4 in H1299 and A549 cells suggesting solamargine regulated EP4 at both transcriptional and translational levels (Fig. 3a-c).

### Solamargine inhibited SP1 protein through inactivation of Akt; exogenous expressed SP1 abrogated the effect of solamargine on EP4 expression

**Fig. 4:**
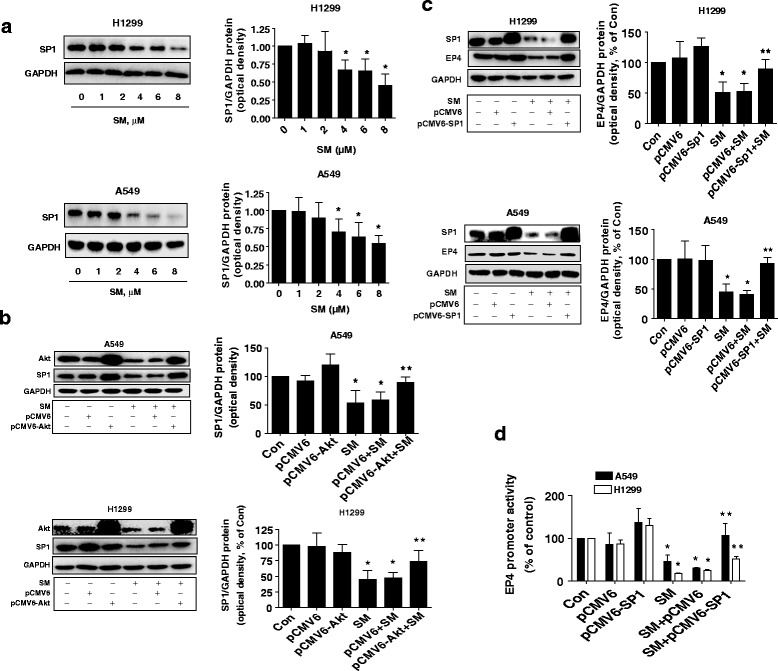
Solamargine inhibited SP1 protein expression through inactivation of Akt; exogenous expression of SP1 abrogated the effect of solamargine on EP4 expression. **a** A549 and H1975 cells were treated with increased concentrations of solamargine for 24 h. Afterwards, the expression of SP1 proteins was detected by Western blot. **b** A549 and H1975 cells were transfected with the control or expression construct of Akt vectors for 24 h before exposing the cells to solamargine for an additional 24 h. Afterwards, the expression of Akt and SP1 proteins were determined by Western blot, and was expressed as percentage of control in the mean ± SD of three separate experiments. GAPDH was used as loading control. **c** A549 and H1975 cells were transfected with the control or expression constructs of SP1 for 24 h before exposing the cells to solamargine for an additional 24 h. Afterwards, the expression of SP1 and EP4 proteins were determined by Western Blot, and was expressed as percentage of control in the mean ± SD of three separate experiments. GAPDH was used as internal control. **d** A549 and H1975 cells were transfected with the control or expression construct of SP1 and wild type EP4 promoter reporter construct ligated to luciferase reporter gene and internal control for 24 h before exposing the cells to solamargine for an additional 24 h. Afterwards, the promoter activities were determined using the Dual-Luciferase Reporter kit (Promega). Values in bar graphs were given as the mean ± SD from three independent experiments performed in triplicate. *Indicates significant difference as compared to the untreated control group (*P* < 0.05). **Indicates significant difference from the solamargine treated alone group (*P* < 0.01)

Moreover, we tested the role of transcription factor SP1 in this process. Previous studies found that EP4 promoter contain SP1 binding sites and that SP1 regulated the function and expression of EP4 [26]. Herein, we showed that solamargine inhibited SP1 protein expression, which was abrogated in cells transfected with exogenous expressed Akt in H1299 and A549 cells (Fig. 4a-b). As expected, exogenous expression of SP1 overcame the effect of solamargine on EP4 protein expression and promoter activity in H1299 and A549 cells (Fig. 4c-d). These results above suggested that SP1 is a downstream of Akt but an upstream of EP4, and regulated EP4 expression both at transcriptional and translational levels.

### Solamargine inhibited p65 protein expression; exogenous expressed p65 reversed solamargine-inhibited EP4 expression

**Fig. 5:**
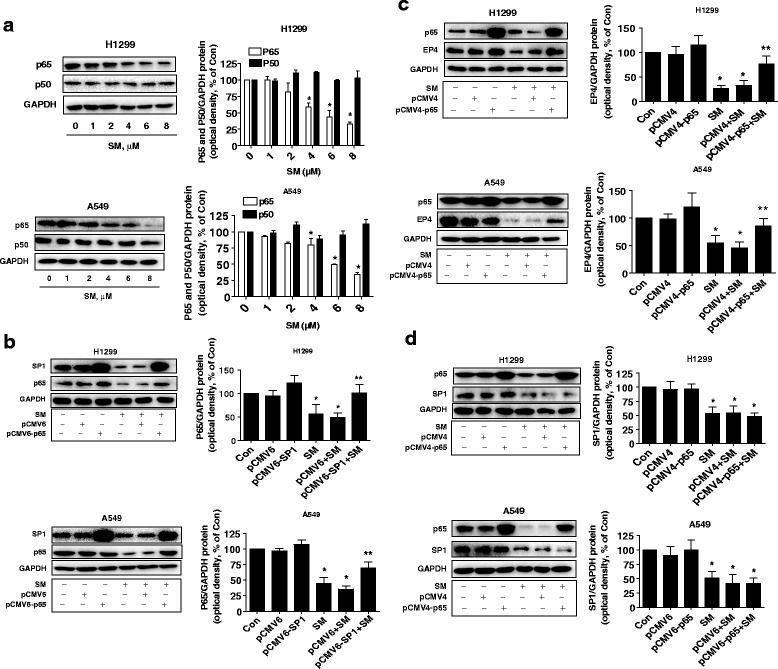
Solamargine inhibited p65 protein expression; exogenous expressed p65 reversed the solamargine-inhibited EP4 expression. **a** A549 and H1975 cells were treated with increased concentration of solamargine for 24 h. Afterwards, the expression of p65 protein was detected by Western blot. **b** A549 and H1975 cells were transfected with the control or expression constructs of SP1 for 24 h before exposing the cells to solamargine for an additional 24 h. Afterwards, SP1 and p65 protein expression were determined by Western blot. **c**-**d** A549 and H1975 cells were transfected with the control and p65 expression vectors for 24 h before exposing the cells to solamargine for an additional 24 h. Afterwards, the p65, SP1 and EP4 protein were determined using Western blot. GAPDH was used as internal control. Values in bar graphs were given as the mean ± SD from three independent experiments performed in triplicate. *Indicates significant difference as compared to the untreated control group (*P* < 0.05). **Indicates significant difference from the solamargine treated alone group (*P* < 0.01)

We also characterized the additional transcription factor NF-κB/p65 in this process. NF-κB signaling has been associated with PGE_2_/EP4 pathway in other studies [27, 28]. To this end, we showed that solamargine inhibited p65 protein expression in H1299 and A549 cells (Fig. 5a), which was not observed in the cells transfected with exogenous expressed SP1 (Fig. 5b). Interestingly, overexpressed p65 reversed solamargine-inhibited EP4 protein expression (Fig. 5c). Of note, exogenous expressed p65 had no effect on solamargine-inhibited SP1 protein expression in H1299 and A549 cells (Fig. 5d) suggesting the downstream effector of SP1 but upstream signal of EP4 in this process.

### Overexpressed EP4 feedback antagonized the effect of solamargine on phosphorylation of Akt and cell growth inhibition

**Fig. 6:**
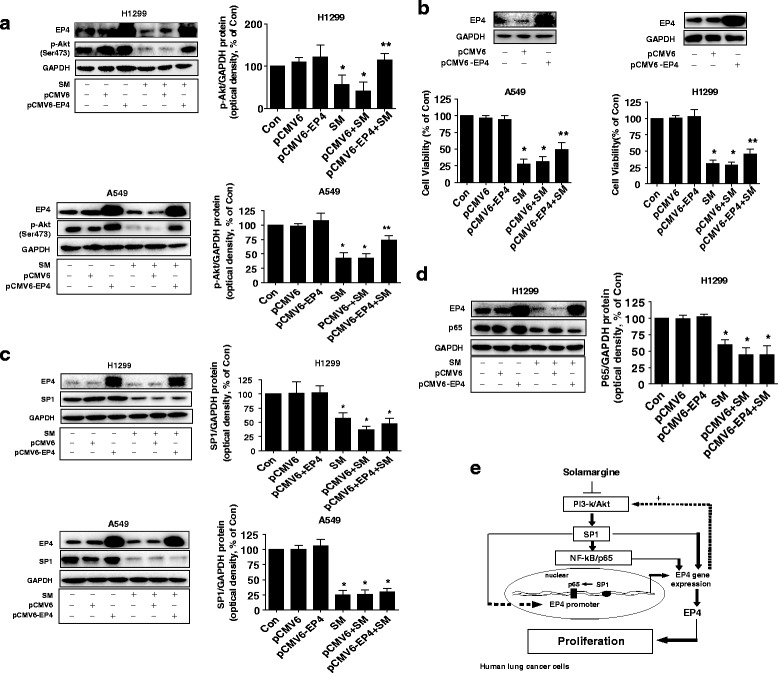
Overexpressed EP4 feedback antagonized the effect of solamargine on phosphorylation of Akt and cell growth inhibition. **a** A549 and H1299 cells were transfected with control and EP4 overexpression vectors for 24 h before exposing the cells to solamargine (6 μM) for an additional 2 h and 24 h. Afterwards, phosphorylation of Akt and EP4 protein expressions were determined by Western blot. **b** A549 and H1975 cells were transfected with control and EP4 overexpression vectors for 24 h before exposing the cells to solamargine (6 μM) for an additional 24 h. Afterwards, EP4 protein expressions and cell viability were determined using Western blot and MTT assays, respectively. Values in bar graphs were given as the mean ± SD from three independent experiments performed in triplicate. *Indicates significant difference as compared to the untreated control group (*P* < 0.05). **Indicates significant difference from the solamargine or plus control vector group (*P* < 0.01). **c**-**d** A549 and H1299 cells were transfected with control (pCMV6) and EP4 overexpression vector for 24 h before exposing the cells to solamargine (6 μM) for an additional 24 h. Afterwards, expressions of SP1, p65 and EP4 protein were determined by Western blot. **e** The diagram the shows that solamargine inhibits the growth of lung cancer cells through inactivation of Akt, followed by reduction of SP1 and p65. This results in the inhibition of expression of EP4 gene*.* The cross-talk between SP1 and p65, and the positive feedback regulatory loop of PI3-K/Akt signaling by EP4 contribute to the overall responses of solamargine in this process

Finally, to further corroborate the critical role of EP4 in this process, we transfected with exogenous expressed EP4 plasmid into the cells. The results indicated that overexpression of EP4 feedback antagonized solamargine-inhibited phosphorylation of Akt (Fig. 6a) and, more importantly, significantly blocked the effect of solamargine on cell growth inhibition (Fig. 6b). Note that overexpression of EP4 had no effect on solamargine-reduced SP1 and p65 protein expression (Fig. 6c-d). This result unveils a novel feedback regulatory loop and suggested that SP1 and p65 are both upstream molecules of EP4.

## Discussion

Lung cancer still remains a major threat to public health worldwide, the incidence and motility rates have been increased continuously. In spite of the advance in understanding the molecular biology and therapeutic modalities, less progress has been made in improving quality of life and survival in patients with advanced stage NSCLC [1, 29]. Therefore, developing new adjunctive therapeutics to augment currently available treatment modalities without compromising therapeutic efficacy are eagerly needed.

Solamargine, a steroidal alkaloid glycoside extracted from the traditional Chinese herb *Solanum incanum*, has been shown to inhibit growth and induce apoptosis in various cancer types including lung through multiple signaling pathways and mechanisms [4, 5, 30]. However, the detailed molecular mechanism underlying inhibition of proliferation of lung cancer cells still remain to be elucidated. In this study, we demonstrated a significant inhibition of lung cancer cell growth by solamargine. The doses of solamargine examined in the current study were similar or even lower than those reported by others demonstrating substantial growth inhibition in several cancer cell types [4, 30, 31].

In this study, we explored the potential mechanism by which solamargine inhibited NSCLC cell growth. Our results indicated a role of PI3-K/Akt signaling pathway in mediating the effect of solamargine in inhibition of lung cancer cell growth, suggesting that inactivation of PI3-K/Akt increased the effect of solamargine in this process. The activation of PI3-K/Akt axis implicates in the regulation of other gene expression and cellular responses in various types of cancer including lung, therefore, approaches to harbor this signaling axis could be valuable potential for treating cancers [32, 33]. Because of the fact that less or no data have shown the links between the solamargine and PI3-K/Akt, we believed that blockade of this signaling pathway could be part of the anti-tumor mechanism of solamargine.

To further explore the potential mechanism underlying the aforementioned, we tested the role of EP4, one of the PGE_2_ receptor, which was involved in proliferation, migration and invasion of cancer cells [19]. Our results indicated the critical role of EP4 involved in the effect of solamargine on inhibition of lung cancer cell growth, implying that EP4 could be a potential target in the treatment of lung cancer. Recent studies showed that inhibition of EP4 suppressed growth of several cancer cell types emphasizing the important role of this prostanoid receptor [34, 35]. We believed that, as unfavorable tumor-promoting role, EP4 could be a new target in mediating the inhibitory effect of solamargine in lung cancer intervention.

Furthermore, we found a causative role of transcription factor SP1 that involved in the effects of solamargine on suppression of EP4 expression and lung cancer cell proliferation. Our findings indicated that inhibition of SP1 was required to enhance the effect of solamargine on inhibition of EP4 receptor expression. The links of EP4 and SP1 signaling have been shown in other studies [22, 26]. Early study demonstrated that human EP4 gene promoter contained SP1 DNA binding sites and regulated EP4 expression [26]. Overexpression of SP1 has been shown to overcome the inhibitory effect of curcumin on EP4 promoter activity and protein expression in other cancer cell types [22]. Thus, we reasoned that regulation of SP1 played a crucial role in mediating solamargine-inhibited EP4 expression in lung cancer cells.

We also observed the involvement of PI3-K/Akt signaling pathway in the regulation of SP1 and EP4 in response of solamargine-inhibited lung cancer cell growth. The role of PI3-K/Akt signaling in the link of SP1 and EP4 expression and function has been shown in other studies; activation of PI3-K/Akt could alter expression of SP1 and EP4, and thereby influenced differentiation, angiogenesis, metastasis, invasion in several different cell systems [21, 36–38]. Interestingly, we observed a positive feedback regulatory axis of PI3-K/Akt by EP4. We reasoned that this bidirectional feedback loop might also implicate in the solamargine-inhibited lung cancer signaling. The feedback regulation circuit of PI3-K/Akt with other targets was reported in other studies demonstrating common autocrine physiopathological phenomena [39–41]. One study showed that NADPH oxidase 4 (NOX4) was highly expressed in NSCLC cells and there existed a mutual positive regulatory loop between NOX4 and PI3-K/Akt signaling in NSCLC cells that contributed to proliferation and progression [42]. Nevertheless, more experiments are needed to better elucidate the in-depth mechanism such as the potential signaling pathways and up- or downstream mediators that may be involved in this unexplored regulatory circuit.

Moreover, our results illustrated the cross-talk between the SP1 and NF-κB/p65 that contributed to the solamargine-reduced EP4 expression in lung cancer cells. Previous studies found that EP4 promoter contain several putative DNA binding sequences including SP1, which could influence the function and expression of EP4 [26]. Our findings indicated the upstream role of SP1 in regulating the solamargine-reduced EP4 expression. Moreover, we also demonstrated the interaction between SP1 and p65, one subunit of NF-κB, suggesting that inter-correlation of the transcription factors cooperatively contributed to the inhibitory effects of solamargine on EP4 expression at both transcriptional and translational levels. Accumulating evidence indicated that deregulation and inter-collaboration of SP1 and NF-κB/p65 expression was implicated in the several pathological responses, such as growth and progression of many cancer types, by affecting the expression of other target genes [43–47] The critical roles of these transcription factors in influencing the PGE2/EP4 signaling and relevant connections-mediated functions were reported in other studies [22, 28, 48, 49]. More experiments are required to determine if there is a physical binding between SP1 and NF-κB/p65 that might regulate solamargine-suppressed EP4 gene expression and subsequent cell growth inhibition. In addition, whether this is a direct or indirect interaction with other DNA sequences in the EP4 gene promoter remains to be elucidated. We believed that understanding the complex oncogenic network is critical for identifying therapeutic targets for lung cancer therapy. [50, 51]. Nevertheless, the precise mechanism underlying this intercommunication in influencing EP4 expression required to be determined.

## Conclusion

Collectively, our results show that solamargine inhibits the growth of lung cancer cells through inactivation of Akt, followed by reduction of SP1 and p65. This results in the inhibition of expression of EP4 gene*.* The cross-talk between SP1 and p65, and the positive feedback regulatory loop of PI3-K/Akt signaling by EP4 contribute to the overall responses of solamargine in this process (Fig. 6e). This study unveils the novel mechanism by which solamargine inhibits growth of human lung cancer cells and reemphasizes the potential target of EP4 in lung cancer prevention and treatment.

## Abbreviations

NSCLC: Non small cell lung cancer
PI3-K: Phosphatidylinositol 3-kinase
qRT-PCR: Quantitative real-time PCR
SM: Solamargine
SL: Solanum lycocarpum
HER2: Human epidermal growth factor receptor 2
TOP2A: Topoisomerase IIalpha
Akt: Protein kinase B
TKIs: Tyrosine kinase inhibitors
PGE_2_: Prostaglandin E_2_
EGF-R: Epidermal growth factor receptor
mTOR: Mammalian target of rapamycin
NOX4: NADPH oxidase 4
EP4: E-type prostanoid 4
MTT: 3-(4, 5-dimethylthiazol-2-yl)-2, 5-diphenyltetrazolium bromide
